# The Rights of Authors

**Published:** 1857-12

**Authors:** Martyn Paine

**Affiliations:** Prof. of General Therapeutics and Mat. Med. in the University of New York


### SELECTIONS.

*The Rights of Authors.* By MARTYN PAINE, A. M., M. D., Prof. of General Therapeutics and Mat. Med. in the University of New York.

[The Editors of the *Medical Independent* will much oblige Dr. Paine by inserting in the journal the following extract from an *Appendix* to his "*Institutes of Medicine,*" which is now in the press.]

Upon all questions of priority that concern the advancement of Science and Art, there is, doubtless, a general understanding that the principle should not only be sacredly observed, but that, whenever violated, there should be a common effort to repair the injury. This is alike due to the individual, to the principle, and to the common good. Nor is it less the privilege of the individual, who may have good reason to think that the principle has not been extended to himself, to vindicate his rights, and to appeal to that sympathy which forms the bond of union among honorable men. It is a common cause, and not seldom demands protection.

The author of these Institutes (and it will soon appear that he acted wisely) has sometimes thought it expedient to assert his claim of originality, in advance, to many doctrines promulgated in the word; as, for example, all that is most essential in the application of the nervous power, or reflex action of the nervous system to pathology, and therapeutics, and so much of what is most important in the natural state of the function. This may be readily seen by consulting p. 106, § 222 *b*, p. 107–116, § 225–234, p. 295, § 476 *a*. p. 318, § 493 *d*, p. 321, § 496–497, p. 323–340, § 500–514, p. 465–467, § 714–719, p. 506, § 803–804, p. 515–516, § 819 *b*, p. 661–663, § 894–896, p. 666–676, § 902 *b*–904, p. 679–680 § 905, *a*, p. 690–691, § 906 *g*, p. 693–695, § 917–923, p. 698, § 931–935, p. 703–711, § 940–952, p. 746, § 990 1-2 *b*, etc., where all the subjects relate to the reflex action of the nervous system, and present the nervous power as an important vital agent in the various processes of organic and animal life, in the production of disease, in the operation of remedies, in all the results of bloodletting, in the changes which take place in the secreted and excreted products—having also originally set forth the agency of the nervous power in voluntary motion Index I., Article, *Will*), and as this power is the efficient cause of the modifications of organic results under the influence of mental emotions (Index) I., Article, *Mental Emotions*, and references at p. 867, § 1067).

Indeed, as the reader will have seen the foregoing doctrines relative to the reflex action of the nervous power, operating as a vital *stimulus*, or vital *pepressant*, or vital *alterative*, as it may be modified in its nature by one cause or another (§ 107–109, 227–230, p, 661–662, § 894 *b*), pervade this work. The same doctrines are at the foundation of the author's "*Medical and Physiological Commentaries*," published in 1840, while the present work was published in 1847. In the mean time he has also labored to inculcate them thoughout his course of medical lectures in the University of New York—first on the institutes of medicine and materia medica from the year 1841 to 1850, and subsequently, to the present time (1857), on general therapeutics and materia medica.

It may be worth saying also, that the author preserves the term "sympathy," though always meaning by it, as he strictly defines, reflex action of the nervous system, and this whether he employs the term "remote sympathy" or "contiguous sympathy." The elements of sympathy, as set forth in the work, are the *nervous power* and *sensibility*. All this will be readily seen by reference to Index I. Also among other general remarks of a similar import, the author has the following:

" Notwithstandding all the laws of sympathy that are necessary to the full interpretation of the remote effects of morbific and remedial agents are as well established as any laws in physics, *they have not been applied to these important objects;* but on the contrary, those philosophers who have contributed most to their critical exposition, *overlook their pathological and therapeutical bearings*, *and cling to the doctrines of humoralism and of the operation of remedies by absorption; nor have they applied, in the least*, *the nervous power in a philosophical manner to an explanation of the natural phenomena of sympathy* (p.111, § 234 a).

When the foregoiong works were first published, it was in the midst of a universal prevalence of the chemical and physical doctrines of life and disease, and the author stood alone in the field of vital physiology, and in the application of the reflex action of the nervous system in resolving the problems in physiology, and therapeutics. A few, however, had the quick sagacity to see its importance as presented by the author; and since the decline of organic chemistry began, others have entered upon the inquiry and the most zealous have promulgated as original with themselves many of the doctrines which belong to the author of these Institutes, especially such as are relative to the nervous system. But the author has relied upon his prfessional brethren for ultimate justice: "*Ultimum et muicum remedium.*" *Jus aliquando dormitur*, *moritur nunquam.*"

But the author has lately seen so great an indisposition, in certain quarters, to allow him any credit for his labors, that he has concluded to make this expostulation, which refers, particularly, to the following dispute about the authorship of matters in which neither of the gentlemen has any interest, but the writer alone of these Institutes. This rival claim appears in an article published by J. ADAMS ALLEN, A. M., M. D., in the "*Medical Independent*" for September, 1857, p. 381, Detroit, Michigan. Thus:

"It appears from a late number of the *London Lancet* that M. Hall, (Marshall Hall) recognizes, to a certain extent, the priority of Dr. Campbell. His words are these:

"I arrive at this conclusion: the idea and the designation of an excito-*secretory* action belongs to Dr. Campbell, and his details are limited to pathology and observation. The elaborate experimental demonstration of reflex excito-secretory action is the result of the labors of M. Claude Bernard. My own claim is of a very different character, and I renounce every other. It consists in the vast *generalization* of excito-secretory action throughout the system. I trust Dr. Campbell will be satisfied with my adjudication. There is in the excito-secretory function as applied to pathology, an ample field of inquiry for his life's career, and it is indisputably—HIS OWN. He first detected it, gave it its designation, and saw its vast importance."

Dr. ALLEN then continues:

"M. Hall thus far freely and fully admits the priority of Dr. Campbell, and the latter gentleman bases his claim upon the date, May, 1850. I shall undertake to show that this same doctrine was FIRST publicly announced and illustrated in my lectures at the Indiana Medical College, in November, 1848, and thenceforth continuously during the continuance of my public teaching before the several classes of the University of Michigan until my connection with that Institution 'expired by limitation' in 1854. My own manuscript containing this doctrine was written in May or June, 1848,"—that is to say, *more than one year after the publication of these Institutes.*

"What I do claim *is the great generalization that the excito-influence is followed by a reflex change in which the effect is* NOT A MOTION *but* A MODIFICATION OF VASCULAR AND NUTRIENT ACTION. *That this effect takes place* BY MEANS OF THE DOUBLE NERVOUS ARC. A vast number of therapeutic phenomena are thus explained." [As the reader will find, very extensively, in many chapters of this work, particularly in those upon REMEDIAL ACTION, THERAPEUTICS, COUNTER-IRRITATION, CATHARTICS, ETC.—Dr. A.'s capitals and italics.]

Now the whole of the foregoing doctrine is impressed upon the *Medical and Physiological Commentaries*, and has been always taught extensively in the author's lectures since 1841.

Dr. ALLEN claims, also, the application of the principle to therapeutics, and remarks that "in my course upon 'general therapeutics' the subject of counter-irritation ' came under review," and concludes that "the impression MUST BE transmitted to the nervous centres, and thence reflected to the affected organ. In other words, the influence is primarily exerted upon the cerebro-spinal system, and secondarily upon the internal affected organ. This is the gist of the whole matter, and the point consists in the recognition of reflex cerebro-spinal action, which, in the instances adduced, give rise to a *molecular* or *integral* change in the inflamed tissue, and not a muscular contraction. The oral elaboration o this principle was suggested by an idea (?) which does not even now appear to have occurred to either M. Hall or Dr. Campbell, viz: The motor effect is merely *secondary*, and not a necessary part of the action of the nervous arc."

Here, also, the whole of the foregoing doctrine appears throughout these institutes. But they embrace a long chapter, particularly upon "*counter-irritation*," in which it will be seen that the author has employed nearly the foregoing language of Dr. ALLEN, especially at pages 646, 647, § 893 *e*, and with great elaboration and extensive application of the doctrine throughout the work; which had been also antecedently taught in his lectures for seven consecutive years before Dr. ALLEN promulgated the same views.

To show still farther this partiality for the Author's writings, or his lectures (then familiar to his large classes of students,) he will quote from Dr. ALLEN the following conclusions, which he also places in capitals:

"THE EFFECT IS MOTORY, IF CONTRACTILE FIBRE BE PRESENT.

"THE EFFECT IS SECRETORY, IF SECRETORY ORGANS BE SUPPLIED.

"THE EFFECT IS SENSATION, IF SENSITIVE NEURINE BE REACHED.

"THE EFFECT IS PERCEPTION, OR INTELFECTION, IF THE ORGAN THEREOF BE IN CONNECTION WITH THE REFLEX NERVE."

"The effect produced, then, depends upon the *structure and condition* of the organ reached."

"This influence is *not* confined to mere increase of action, as the term EXCITOR might perhaps suggest. The reverse may take place—the excitor may rather become the *depressor*. It would be as correct to say the excitor-*idem*."

Now, the author of these Institutes not only dwells emphatically upon the *depressing* and *sedative* influence of reflex nervous action, according to the nature of the remote causes and special conditions of disease (p. 107–111, § 226–233 3-4, p. 507, § 806, p. 661–662, § 894–895, p. 671–672, § 904 *a*, p. 735, § 978, and *references* in § 1067 *a*, *b*, as to *mental emotions*, and in many other places,) and upon its operation according to the natural structure and special vital constitution of organs, and their varying conditions (p. 59, § 129 *g–i*, p. 61–69, § 132–156, p. 73, § 163, p. 109, § 229, p. 111, § 233 3–4, p. 285, § 555 *d–f*, p. 313, § 487 *h*, p. 353–361, § 525–529, p. 374––383, § 576–584, p. 415–417, § 649, p. 418, § 651 *b*, p. 421–423, § 657–658, p. 522, § 827 *c*, p. 542, § 854 *bb*, p. 613, § 892 1-2 *b*, p. 644–650, § 893 *c–i*, p. 665–672, § 902–903 *b*, p. 746, § 990 1-2 *b*, and the numerous references in those sections ;) but the Author represents, also, the reflex action as variously *alterative* in organic life, and this imputed attribute pervades the author's writings. He enforces, everywhere, the doctrine that the reflex action of the nervous power is the modifying cause through which all the changes are effected by morbific and remedial agents in parts that are not immediately connected with the direct seat of their action ; and, farther, that the principle is precisely the same when the nervous power is brought into operation by *direct* influences upon the nervous centres (as in the case of their diseases, or when the passions operate, or as the will determines voluntary motion,) as it is when it is brought into operation in that indirect manner known as reflex action.

Indeed, every one of the foregoing doctrines, in all their particularities, as quoted from the American claimant, are taught, at great extent, in the volume before us, as may be readily seen by consulting the references made in this protest, and more extensively, Index I., Articles STRUCTURE, NERVOUS POWER, SENSATION, SENSIBILITY, SYMPATHY, ORGANIC FUNCTIONS, REMEDIAL ACTION, MIND, MENTAL EMOTIONS, WILL. "*Si quæris monumentum, circumspice.*" It may appear superfluous, however, to have made these specific references in an article connected with the work itself ; but it is done to encourage those readers who might not otherwise be inclined to ascertain the facts.

But the writer is more interested with the European claimants, of whom he has felt that he has much more reason to complain.

> "Omne animi vitium tanto conspectus in se
> Crimen habet, quanto major, qui pecat, habetur."—JUVENAL.

That the author's physiological and medical writings were generally known in Europe many years before the period at which "Dr. Campbell bases his claim" (1850,) is evident from the distinguished honors to which they had [illegible] that country before that period—that from the Medical Society of Prussia as early as 1842—that from the Medical Society of Leipsic in 1843; and the "Medical and Physiological Commentaries" (of 1840) were published simultaneously in London and New York; and as to the United States, the *Commentaries* were early distributed throughout the land, and his *Institutes of Medicine* more than a year, also, before Dr. A.'s lectures were delivered; and the Author's lectures at the University, which form the groundwork of his Institutes, had been listened to annually by medical students from all quarters of the Union since the year 1841. In 1848 the Author applied the doctrine of reflex nervous action to a physiological demonstration of the substantiative existence of the soul and instinctive principle, which was then published in pamphlet form, and in 1849 the work was extended and assumed the shape of a book, and is now incorporated, in its essential parts, in these Institutes.

Nor, is this all; for the whole of this doctrine of reflex nervous action, and of the operation of the nervous power as an *alterative*, an excitant of the secretions and of vascular action (both direct and reflex,) a depressant and sedative (according to the nature of exciting causes,) and the great immediate cause of diseases and their cure—variously modifying organic actions—was set forth extensively and circumstantially in an "Essay on the MODUS OPERANDI OF REMEDIES" in 1842, of which the Author distributed, at that time, a large number of copies in London, and addressed four thousand copies to physicians throughout the United States. The Author not only sent a copy of the work to Dr. Hall, but dedicated it to him (along with Prof. J. Muller and Dr. A. P. W. Philip,) in connection with an "Essay on the Philosophy of Vitality;" and he may add that he controverted, in the former essay, doctrines of Dr. Hall (in "Memoir on Diseases and Derangements of the Nervous System, 1841" which were in direct opposition to those which are now in question (also, p. 296–297, § 476 1-2 *b*.) These Essays were subsequently bound up in the third volume of the "Medical and Physiological Commentaries," where the former may be readily consulted. But Dr. Philip had fully deduced from his experiments the sedative as well as exciting influence of the nervous system upon vascular action before Dr. Hall's experiments were made (§ 492.)

As to M. Bernard, his experiments bearing upon the connection of the nerves with the functions of secretion, however much they may have been varied and multiplied, were anticipated long before by A. W. P. Philip, which are quoted extensively in these Institutes (p. 290–321,)

and towards which Dr. Hall had no friendly disposition (p. 306–308, and where the writer has controverted his views.) The merit of originality which belongs to the present writer, in relation to these experiments, consists in their extensive application in illustrating the functions of the nervous power as a vital agent, profoundly interested not only as an "excito-secretory" power, and a modifying cause of all secreted products, nutrition, etc , when diverted from their natural standard, but in deducing from them a universal agency of the reflex action of the nervous system, though "the double nervous arc," in the production and cure of disease, and by which he labored to explode the chemical and physical doctrines as early as 1840. But, that the writer may not be misapprehended, he will say that he endeavored to establish the fact that secretion in animals, as in plants, is conducted by powers implanted in every part, but that it is constantly influenced physiologically, pathologically, and therapeutically, by reflex action of the nervous system.

The writer is very sensible that unaccountable coincidences often present themselves in the development of new thoughts, and in the discovery of hidden things, especially where enduring reputation may be won '*Ubi, mel, ibi, apes.*'"—"*Uno tiene la fama, y otro carda lana.*" But the reader, with these Institutes before him, will quickly find that much that is claimed by Dr. Hall, and all that he has granted to Dr. Campbell, in the foregoing quotation, and, therefore, all that Dr. Allen appropriates to himself,* abounds in this volume, and, in fact, constitutes the life and soul ("zoe kai psuche") of the work, as it does, also, of the "Commentaries," and of the Essay on the "Modus Operandi of Remedies;" nor can the reader fail of the conclusion that, were Dr. Hall's "adjudication," and Dr. Allen's after-thought, founded in any justice, and were not the claimants themselves the obnoxious parties, the present writer would have been long ago convicted by them and by others of arrogant assurance and the grossest plagiarisms. Nevertheless, the Author is most happy to find that his solitary position is becoming relieved, and that a practical direction has been given to his labors by others which cannot fail of carrying forward the great doctrines at which he has toiled, and against manifold obstacles, during his professional life.—*Med. Indep.*

NEW YORK, Sept., 1857.

---

* "Unus utrique
Error; sed variis illudit partibus."—HORACE.

